# Accuracy and Usability of Emergency Department Diagnostic Codes: A Multicentre Observational Study

**DOI:** 10.3390/diagnostics16172872

**Published:** 2026-09-07

**Authors:** Viet Tran, Elizabeth Tham, Simone Page, Lauren Thurlow, Lizette Tredoux, Giles Barrington

**Affiliations:** 1 Emergency Department, Royal Hobart Hospital, Hobart, TAS 7000, Australia; 2 Tasmanian School of Medicine, University of Tasmania, Hobart, TAS 7000, Australia; 3 Menzies Institute for Medical Research, University of Tasmania, Hobart, TAS 7000, Australia; 4 Tasmanian Emergency Medicine Research Institute, Hobart, TAS 7000, Australia

**Keywords:** emergency medicine, diagnostic coding, health services research

## Abstract

**Objectives:** To evaluate the accuracy and usability of emergency department (ED) clinician-assigned diagnostic codes across all four Tasmanian public hospitals. **Methods:** We conducted a retrospective study of 300 ED presentations from all four public EDs in Tasmania. Two reviewers, a specialist emergency physician and a clinical research fellow, classified the accuracy of diagnostic codes across five levels which were then further classified into usable (levels 1–2) and unusable (levels 3–5). Rater agreement was evaluated. For accuracy and usability evaluation, conflicting reviewer classifications were removed. Associations between usability and patient, presentation, and site characteristics were evaluated using chi-square and Fisher’s exact tests. **Results:** Overall rater agreement was moderate for both accuracy (69.3%, κ = 0.52) and usability (81.7%, κ = 0.58). Among 208 eligible presentations, 139 (66.8%) diagnostic codes were correct, 153 (73.6%) were acceptable for secondary use, 9 (4.3%) were clearly incorrect and 13 (6.3%) had insufficient documentation. Coding performance varied across hospitals, acuity, arrival mode, and representation intervals, with hospital-level usability ranging from 63.6% to 88.9%. **Conclusions:** Across all four Tasmanian public EDs, 66.8% of diagnostic codes were accurate, 73.6% were appropriate for secondary use, and 4.3% were clearly incorrect, with moderate rater agreement for accuracy and usability (κ = 0.52 and κ = 0.58). These findings, together with marked between-hospital variation, underscore the context dependence and subjectivity of ED diagnostic coding. They support a cautious approach to using ED diagnostic codes for health service planning, performance reporting, and observational research, while highlighting the need to address barriers and opportunities to strengthen coding performance.

## 1. Introduction

All patients who present to the emergency department (ED) are assigned a diagnostic code that represents the principal diagnosis, the condition identified at the conclusion of an emergency care episode as primarily responsible for the patient’s attendance [1]. Accurate diagnostic coding in EDs is crucial for effective patient care, resource allocation, audit, research, health system management and clinical quality registries including the Tasmanian Emergency Care Outcomes Registry [2,3,4,5,6,7,8,9]. They also directly inform funding in many EDs across the world that operate under activity-based funding models [8]. Understanding the accuracy and usability of these diagnostic codes is therefore important, as inaccuracies can lead to misinformed funding allocations, misleading audit data, and less valid research results [10].

In most jurisdictions, ED diagnostic codes are allocated by clinicians during an episode of clinical care rather than by clinical coders, who are professionally trained specialists and whose sole role is to translate clinical documentation into standardised diagnostic codes [4]. Emergency department clinicians are often not formally trained in effective coding, with one survey indicating that 76% had no formal introduction to the coding system, and 86% reported that multiple diagnostic codes could be appropriate due to overlapping options for discharge code assignment [4].

In terms of performance, diagnostic coding accuracy by clinical coders has been variably reported in the literature from 82.2 to 86.5% accurate [2,11,12]. Similar results were found when ED clinicians were responsible for coding with accuracy from 81.0% to 97.4% [13,14].

Australia’s federated system further complicates diagnostic coding. The Australian Institute of Health and Welfare (AIHW) reports on national ED data, including diagnostic codes based on the Emergency Principal Diagnosis (EPD) short list. However, at the service level, most health services continue to rely on legacy coding systems [1,15,16]. Mapping legacy coding to the EPD short list brings with it additional complexity, especially for some systems such as Systematised Nomenclature of Medicine–Clinical Terms (SNOMED-CT) where substantial use of non-specific, duplicate and redundant codes was found [17]. This in turn erodes the usability of these codes.

Diagnostic codes play a central role in observational research and audit by enabling the identification of specific patient populations and cases for evaluation [18]. Usable diagnostic coding is therefore critical for ensuring that study populations are correctly identified, that comparisons between services or time periods are valid, and that performance metrics reflect true clinical activity rather than coding artefacts [19]. Although there are a handful of studies evaluating the accuracy of diagnostic codes, to our knowledge, no study has ever evaluated the usability of ED diagnostic codes [2,13,20].

The primary aim of this study was to evaluate the accuracy and usability of ED diagnostic codes across all four public EDs in Tasmania, Australia. Secondary aims include evaluating rater agreement, the relationship between accuracy and usability with sex, admission status, representation (48 h, 7 days and 28 days), Australian Triage Scale (ATS, 1–5), arrival mode and hospital.

## 2. Materials and Methods

### 2.1. Setting

This retrospective observational study included patient presentations from all four Tasmanian public EDs. Tasmania has a population of approximately 576,700 people [21]. Based on the Modified Monash Model (MMM) (Australian Government, Canberra, Australia) remoteness scale, all four EDs are located outside metropolitan and major cities with two classified as MMM2 (regional centres) and two classified as MMM3 (large rural towns) [6].

The ED diagnostic code list used in Tasmania was derived from the International Statistical Classification of Diseases and Related Health Problems, Tenth Revision, Australian Modification (ICD-10-AM) (Australian Government, Canberra, Australia) with refinements developed by a statewide ED electronic medical record working group [22]. Diagnostic codes are assigned by clinicians (doctors, nurses, nurse practitioners and physiotherapists) during the patient care episode, with any clinician able to assign a diagnostic code to any patient (including ones they have not cared for). The medical record system does not record who assigns a diagnostic code to a patient. In all four EDs, assigning a diagnostic code before discharge is mandatory within the ED information system.

### 2.2. Design

A blinded dual-reviewer methodology was employed to analyse 300 patient records. The only previous study with the same study design employed a single reviewer to assess all charts, with a second reviewer evaluating only 7.1% of the same charts to estimate inter-rater reliability [13]. We chose to adopt a dual-reviewer methodology to enable rigorous assessment of usability rather than diagnostic accuracy alone. Both reviewers possessed distinct expertise with reviewer one a Fellow of the Australasian College for Emergency Medicine and reviewer two a clinical (nursing) research fellow based in the ED. Our sample size was based on both feasibility of chart review and prior studies showing assessment for accuracy stabilising after just 29 presentations [13]. For each episode of patient care, reviewers were required to assign a level of accuracy for the diagnostic code assigned (Table 1). Accuracy definitions were based on prior studies and modified to reflect usability definitions developed locally through grouping of accuracy levels [13]. Using these definitions, usability included diagnoses that were the most appropriate as well as those that were specific enough to allow for secondary use activities such as auditing, research and service planning. Disagreements were not adjudicated, reflecting the pragmatic reality of clinician-led ED coding where diagnostic uncertainty and code-choice variation are expected [23]. For the analyses of accuracy of reviewer-agreed ED discharge diagnostic codes, only presentations with exact agreement between both reviewers on the five-level accuracy classification will be included; usability will be derived within this same cohort by grouping Levels 1–2 as usable and Levels 3–5 as unusable. Additional data variables collected included sex, admission status, representation (48 h, 7 days and 28 days), Australian Triage Scale (ATS, 1–5), arrival mode and hospital.

The first 300 ED presentations between 1 and 7 January 2024 (inclusive), during the hours 12:00–18:00, were selected. This window was chosen as a pragmatic sampling strategy taking into account a time period with the highest number of different clinicians likely to be working in the EDs. This time frame also served to capture patients seen by both day staff and evening staff with variable degrees of fatigue at the time of recording a discharge diagnosis. Records indicating no true ED presentation were excluded and replaced by the next presentation in the sequence.

### 2.3. Statistical Analysis

We followed the Guidelines for Reporting Reliability and Agreement Studies (GRRAS) for reporting accuracy (Supplementary Materials) [24]. Rater agreement rather than inter-rater reliability was evaluated because there is no established gold standard for what constitutes a correct diagnosis in most cases, against which rater judgement could be validated. Agreement was reported as counts and percentages. For ED diagnostic code accuracy, quadratic-weighted Cohen’s Kappa was used across all levels, and unweighted Kappa for usability. We used the original interpretation of Kappa values to assess degree of agreement (κ < 0 poor; 0.00–0.20 slight; 0.21–0.40 fair; 0.41–0.60 moderate; 0.61–0.80 substantial; 0.81–1.00 almost perfect) [25]. For accuracy and usability analysis, conflicting reviewer code assignments were removed. Categorical variables for accuracy and usability analysis were presented as counts and percentages. Accuracy and usability analyses were also cross-tabulated by each variable. Binominal variables were analysed using Pearson’s chi-square test of independence, and where cell counts were small Fisher’s exact test was used to assess association. For variables with more than two categories, the *p*-value represents the overall test of association for the variable as a whole. Statistical significance was set at *p* < 0.05.

### 2.4. Ethics Approval

This study was approved by the Tasmanian Department of Health Low Risk Human Research Ethics Committee (2024/ETH02902).

## 3. Results

### 3.1. Population Characteristics

A total of 300 ED presentations were included with a median age of 42 years (SD 26.6, IQR 23.0–69.0), 149 (49.7%) female and 151 (50.3%) male. Overall, 106 (35.3%) presentations resulted in admissions. Patients were represented from across the state and included Hospital 1 (H1, 114, 38%), Hospital 2 (H2, 88, 29.3%), Hospital 3 (H3, 56, 18.7%) and Hospital 4 (H4, 42, 14.0%).

### 3.2. Rater Agreement

**Figure 1:**
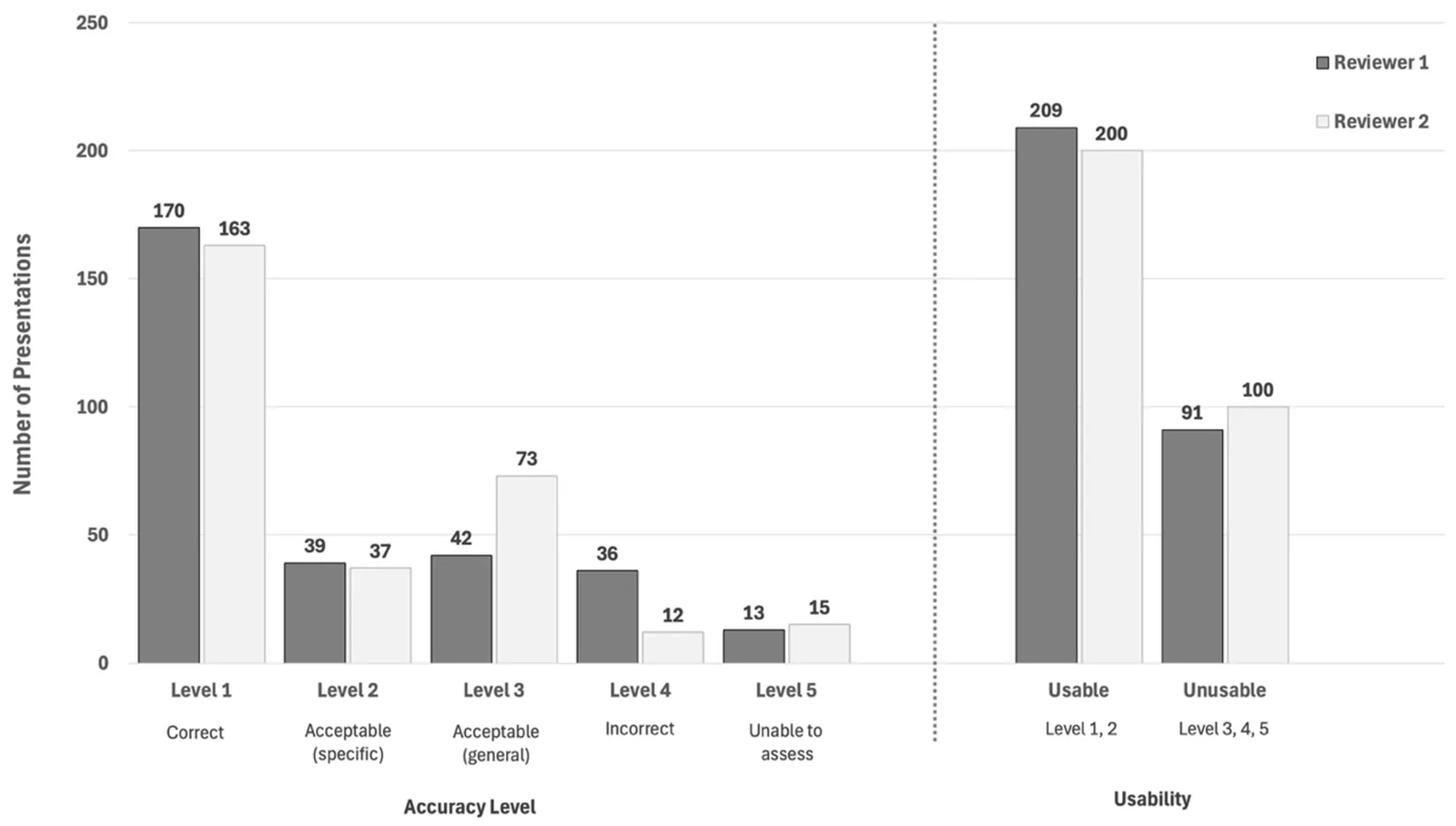
Distribution of rater agreement for accuracy levels and usability of diagnostic codes.

Rater agreement was moderate for both overall usability (81.7%, κ = 0.58) and accuracy (69.3%, κ = 0.52) (Table 2). All variables showed at least moderate agreement apart from accuracy for ATS 5 (66.7%, κ = 0.40). Almost perfect agreement for accuracy was seen for representation less than 48 h, representation less than 28 days (93.8%, κ = 0.89; 86.8%, κ = 0.84) and ATS 1 (66.7%, κ = 0.91). Almost perfect agreement for usability was only seen for ATS 1 (100%, κ = 1.00). For all hospitals, accuracy agreement was substantial (64.3–73.2%, κ = 0.65–0.71) whilst usability agreement was moderate for hospitals 1, 3 and 4 (78.1–85.7%, κ= 0.49–0.56). Hospital 2 had substantial usability agreement (84.1%, κ = 0.63). For accuracy, reviewer two (clinician research fellow) assigned more presentations to level 3 (acceptable—general; 73 versus 42) and fewer to level 4 (incorrect; 36 versus 12) (Figure 1).

### 3.3. Coding Accuracy and Usability

A total of 208 cases (69.3%) had unanimous agreement and were included for analysis of coding accuracy and usability (Table 3). Overall level 1 accuracy was reported in 139 (66.8%) presentations with subgroup accuracy ranging from 50.0% to 88.9%. The five highest performing variables for level 1 accuracy included hospital 4 (88.9%), arrival mode walking (83.8%), ATS 4 (72.4%), representation < 48 h (73.3%) and representation < 7 days (71.0%). An incorrect diagnosis was found in 9 (4.3%) presentations with 8 of those female. Limited documentation that prevented code assessment was found in 13 (6.3%) presentations.

Overall usability was reported in 153 (73.6%) presentations. The five highest statistically significant variables for usability included hospital 4 (88.9%), ATS 2 (69.4%) and 4 (78.2%), hospital 2 (77.8%), hospital 3 (75.6%) and female (73.8%). The lowest statistically significant variable for usability was arrival by a private vehicle (72.9%). Diagnostic code usability between EDs varied, with a statistically significant difference between proportions of usable and unusable diagnostic codes for all hospitals (63.6–88.9, *p* < 0.05).

**Table 3:** Accuracy of reviewer-agreed ED discharge diagnostic codes.

Characteristics	Total *n* (%)	Accuracy Level *n* (%)					Usability *n* (%)		
		1	2	3	4	5	Usable	Unusable	*p*-Value
Total	208 (100.0)	139 (66.8)	14 (6.7)	33 (15.9)	9 (4.3)	13 (6.3)	153 (73.6)	55 (26.4)	<0.001
Female	103 (49.5)	71 (68.9)	5 (4.9)	12 (5.8)	8 (7.8)	7 (6.8)	76 (73.8)	27 (26.2)	<0.001
Admitted	75 (36.1)	45 (60.0)	4 (5.3)	13 (17.3)	4 (5.3)	9 (12.0)	56 (74.7)	18 (25.3)	<0.001
Representation									
<48 h	15 (7.21)	11 (73.3)	0 (0.0)	2 (13.3)	1 (6.7)	1 (6.7)	9 (60.0)	6 (40.0)	0.607
<7 d	31 (14.9)	22 (71.0)	2 (6.5)	4 (12.9)	2 (6.5)	1 (3.2)	20 (64.5)	11 (35.5)	0.150
<28 d	46 (22.1)	30 (65.2)	4 (8.7)	8 (17.4)	2 (4.3)	2 (4.3)	27 (58.7)	19 (41.3)	0.302
Australian Triage Scale									
1	2 (1.0)	1 (50.0)	0 (0.0)	0 (0.0)	1 (50.0)	0 (0.0)	1 (50.0)	1 (50.0)	1.000
2	36 (17.3)	21 (58.3)	4 (11.1)	9 (25.0)	2 (5.6)	0 (0.0)	25 (69.4)	11 (30.6)	0.029
3	81 (38.9)	53 (65.4)	4 (4.9)	16 (19.7)	1 (1.2)	7 (8.6)	57 (70.4)	24 (29.6)	0.003
4	87 (41.8)	63 (72.4)	5 (5.7)	8 (9.2)	5 (5.7)	6 (6.9)	68 (78.2)	19 (21.8)	<0.001
5	2 (1.0)	1 (50.0)	1 (50.0)	0 (0.0)	0 (0.0)	0 (0.0)	2 (100.0)	0 (0.0)	0.500
Arrival mode ^1^									
Private vehicle	96 (46.2)	62 (64.6)	7 (7.3)	16 (16.7)	5 (5.2)	6 (6.3)	70 (72.9)	26 (27.1)	<0.001
Ambulance	60 (28.8)	35 (58.3)	3 (0.5)	13 (21.7)	3 (0.5)	6 (10.0)	44 (73.3)	16 (26.7)	<0.001
Walking	37 (17.8)	31 (83.8)	3 (8.1)	2 (5.4)	1 (2.7)	0 (0.0)	27 (73.0)	10 (27.0)	0.008
Hospital									
Hospital 1	77 (37.0)	44 (57.1)	5 (6.5)	18 (23.4)	3 (3.9)	7 (9.1)	49 (63.6)	28 (36.4)	0.022
Hospital 2	63 (30.3)	43 (68.3)	6 (9.5)	9 (14.3)	2 (3.2)	3 (4.8)	49 (77.8)	14 (22.2)	<0.001
Hospital 3	41 (19.7)	28 (68.3)	3 (7.3)	3 (7.3)	4 (9.8)	3 (7.3)	31 (75.6)	10 (24.4)	0.002
Hospital 4	27 (13.0)	24 (88.9)	0 (0.0)	3 (11.1)	0 (0.0)	0 (0.0)	24 (88.9)	3 (11.1)	<0.001

^1^ Arrival modes with a count of less than 10 were not reported.

## 4. Discussion

To our knowledge, this is the first accuracy and usability assessment of ED diagnostic codes assigned by clinicians in rural and regional contexts. Overall rater agreement was moderate for accuracy (69.3%, κ = 0.52) and usability (81.7%, κ = 0.58). A correct (level 1) diagnostic code was found in 66.8% of presentations whilst a usable diagnostic code (level 1 or 2) was found in 73.6% of presentations. Variables more likely to yield usable diagnostic codes (above 70%, *p* < 0.05) included sex, admission status, ATS 3 and 4 and arrival by private vehicle, ambulance or walking. All hospitals yielded statistically significant results in the usability of their diagnostic codes, with usability ranging from 63.6% to 88.9%.

The identification of 26.4% of diagnostic codes as unusable, together with inter-ED variation, serves as a timely reminder that ED diagnostic codes should be interpreted and applied with caution when used for policy, quality assurance, internal and external benchmarking, service planning, and research [2,11,26,27,28,29]. These concerns are consistent with the literature showing that administrative data validity is context-dependent and coding quality varies by workflow, setting, coder and clinician practice [2,11,22,26].

When compared with the other ED-led coding literature, our accuracy and usability estimates (66.8%, 73.6%) were lower than previously reported studies of accuracy (81.0–97.4%) [13,20,30]. Clinician-entered real-time SNOMED diagnosis recording in a New Zealand tertiary ED showed 97.4% agreement against blinded audit-derived diagnosis labels [20]. This is unsurprising given that SNOMED was created with the aim of providing clinicians with a more intuitive and standardised method for recording diagnoses [17,20]. A single-centre study in Scotland found 81.0% accuracy from 282 presentations but did not evaluate usability [13]. A study in Canada across 11 EDs found an accuracy of 82.2–86.5% although code assignments in these settings were by trained clinical coders rather than clinicians [30]. Our accuracy and usability also fell short compared to the accuracy of clinical coders (66.8% versus 82.2–86.5%). This difference may reflect the clinical coders’ extensive training and primary professional focus on clinical coding, despite not having a clinical background, whereas emergency department clinicians are primarily focused on patient care and coding is not usually a core component of their clinical role [2,11,12].

Regional comparisons found that hospital one accuracy and usability were comparatively lower with an acceptable general diagnosis (level 3) that is not usable for secondary activities assigned in 23.4% of presentations compared with the other EDs (7.3–14.3%). Usability for hospital 1 was 63.6% compared with the other EDs (75.6–88.9%). Although specific ED characteristics including workforce and models of care, were not readily available, hospital one does have an annual census at least twice that of any of the other EDs whilst the other EDs have a higher dependency on locum medical and nursing staff in their EDs. These broad differences do not readily explain the poor performance of hospital one’s accuracy and usability findings and further research is therefore warranted. The variation between hospitals underscores the limitations of unconditional trust in ED diagnostic codes, as rigorous coding may inflate apparent disease burden while broader or less specific coding may create an artificially favourable profile [13].

The ED serves as the principal entry point to care for patients requiring urgent medical attention or those lacking alternative treatment options, encompassing presentations ranging from minor, undifferentiated illnesses to life-threatening conditions necessitating immediate intervention [7]. For most patients, coding a diagnosis is not a priority for the delivery of high-quality care. This is evidenced in the AIHW 2024–25 ED data where the most common principal ICD-10-AM diagnosis group was ‘Symptoms, signs and abnormal clinical and laboratory findings, not elsewhere classified,’ accounting for 27% of all presentations or approximately 2.5 million presentations [16]. For our accuracy assessment, these diagnostic codes should register as level 3 (could be better—difficult to audit) and be considered unusable. Given the AIHW data, it was surprising to find that our study showed a much lower proportion of level 3 (15.9%) diagnostic codes assigned. One reason for this discrepancy likely relates to how data may be mapped from jurisdictional data warehouses with the AIHW data dictionary [17].

Rater agreement in our study was moderate overall for both usability (81.7%, κ = 0.58) and accuracy (69.3%, κ = 0.52). The greatest disagreement between reviewers was for accuracy level 3 with reviewer two assigning 31 (73%) more presentations to this level, followed by accuracy level 4 where reviewer one assigned 24 (66.7%) more presentations. This may be partly explained by reviewer two’s background as a clinical research fellow with expertise in using this data for audit and research whereas reviewer one is a FACEM with significant clinical experience but no additional audit and research skills or experience outside those required during training and as part of continuing professional development.

### Limitations

Limitations included the observational design, which limits causal inference [18]. Our sampling strategy was limited to a fixed time period each day which does not consider the possibility of case mix and coding practices outside these hours. We used a single-blinded technique for data collection, blinding only to the other reviewer and not to the diagnostic code assigned by the clinician during the presentation. This limitation is accepted given similar methodology in prior studies and the need to assess usability of the assigned diagnostic code, something not possible with a double-blinded approach. Our accuracy and usability levels were also reliant on reviewer expertise with usability inferred by reviewers rather than directly tested. This was clear given the rater difference for level 3 accuracy. While the study was multicentred, it reflects a single state with a single ED coding system and therefore may not generalise directly to settings with different coding systems, staffing models, or coding training and cultures. Small counts in some subgroups also constrained precision for some variables. Sample size and the two-reviewer model were additional limitations with bias mitigation discussed in the methodology. The inability to determine the discipline and level of clinicians assigning diagnostic codes also limited interpretation of the results, especially given the between-ED differences found.

## 5. Conclusions

In this multicentre study of four public EDs in Tasmania, we found that clinician-assigned diagnostic codes were 66.8% accurate, 73.6% were usable for audit or research and 4.3% were incorrect. Rater agreement for both accuracy and usability was moderate (69.3%, κ = 0.52; 81.7%, κ = 0.58), underscoring the inherent subjectivity of diagnostic code selection in a specialty where assigning a diagnostic code is not a priority over delivery of high-quality care. The marked variation in accuracy and usability between hospitals, and across triage categories and arrival modes could not be determined from the present study because workflow, staffing characteristics, clinician training, and local coding practices were not directly assessed. These findings have immediate implications for health service planning, performance reporting, and observational research in Tasmania where rigorous coding may inflate apparent disease burden while broader or less specific coding may create an artificially favourable profile. Our results therefore support a cautious approach to interpreting ED diagnostic data. Future work should explore barriers and opportunities to strengthen coding performance.

## Figures and Tables

**Table 1:** Accuracy and usability level descriptions.

Usability	Accuracy Level	Accuracy Short Title	Description
Usable	1	Correct	Most appropriate code
	2	Acceptable—Specific	Acceptable code—code specific enough to allow for secondary use ^1^
Unusable	3	Acceptable—General	Acceptable code—code not specific enough to allow for secondary use ^1^
	4	Incorrect	Inappropriate code used
	5	Unable to assess	Unable to assess due to lack of documentation

^1^ Secondary use includes activities such as auditing, research and service planning.

**Table 2:** Rater agreement profile for accuracy and usability of ED diagnostic codes.

Characteristics	Total *n* (%)	Accuracy *n* (%)	Cohen’s κ	Usability *n* (%)	Cohen’s κ
Total	300	208 (69.3)	0.52	245 (81.7)	0.58
Sex					
Female	149 (49.7)	103 (69.1)	0.69	119 (79.9)	0.94
Male	151 (50.3)	105 (69.5)	0.71	126 (83.4)	
Admission Status					
Admitted	106 (35.3)	75 (70.8)	0.64	85 (80.2)	0.785
Non-admitted	194 (64.7)	133 (68.6)	0.72	160 (82.5)	
Representation					
<48 h	16 (5.3)	15 (93.8)	0.89	14 (87.5)	0.22
<7 d	41(13.7)	31 (75.6)	0.63	24 (82.8)	0.22
<28 d	53 (17.7)	46 (86.8)	0.84	45 (84.9)	0.01
Australian Triage Scale					
1	3 (1.0)	2 (66.7)	0.91	3 (100.0)	0.56
2	52 (17.3)	36 (69.2)	0.66	43 (82.7)	
3	126 (42.0)	81 (64.3)	0.66	96 (76.2)	
4	116 (38.7)	87 (75.0)	0.75	100 (86.2)	
5	3 (1.0)	2 (66.7)	0.40	3 (100.0)	
Arrival mode ^1^					
Private vehicle	139 (46.3)	96 (69.0)	0.70	117 (84.2)	1.00
Ambulance	74 (24.7)	60 (81.1)	0.52	59 (79.7)	
Walking	72 (24.0)	37 (51.4)	0.73	55 (76.4)	
Hospital					
Hospital 1	114 (38.0)	77 (67.5)	0.69	89 (78.1)	0.05
Hospital 2	88 (29.3)	63 (71.6)	0.71	74 (84.1)	
Hospital 3	56 (18.7)	41 (73.2)	0.68	46 (82.1)	
Hospital 4	42 (14.0)	27 (64.3)	0.65	36 (85.7)	

^1^ Arrival modes with a count of less than 10 were not reported.

## Data Availability

The data presented in this study are available upon request from the corresponding author due to local privacy laws.

## Supplementary Materials

The following supporting information can be downloaded at: https://www.mdpi.com/article/10.3390/diagnostics16172872/s1, Table S1: GRRAS checklist for reporting studies of reliability and agreement.
