# Supplementary material for: Accuracy and Usability of Emergency Department Diagnostic Codes: A Multicentre Observational Study
Source: Diagnostics (Basel). 2026 Sep 7;16(17):2872. doi: 10.3390/diagnostics16172872 (PMC13564836; doi:10.3390/diagnostics16172872)
Supplement: Supplementary file 1 [file diagnostics-16-02872-s001.zip › diagnostics-4475546-supplementary.pdf]

Table S1: GRRAS checklist for reporting studies of reliability and agreement

Version based on Table 1 in: Kottner J, Audige L, Brorson S, Donner A, Gajewski BJ, Hróbjartsson A, Roberts C, Shoukri M, Streiner DL. Guidelines for Reporting Reliability and Agreement Studies (GRRAS) were proposed. Int J Nurs Stud. 2011 Jun;48(6):661-71. doi: 10.1016/j.ijnurstu.2011.01.016. Epub 2011 Apr 23. PMID: 21514934.

Accuracy and Usability of Emergency Department Diagnostic Codes: A Multicentre Observational Study

Viet Tran, Elizabeth Tham, Simone Page, Lauren Thurlow, Lizette Tredoux and Giles Barrington
Corresponding Author: Viet Tran, v.tran@utas.edu.au

Section	Item#	Checklist Item	Reported on page #
Title and abstract	1	Identify in title or abstract that interrater/intrarater reliability or agreement was investigated	1
	2	Name and describe the diagnostic or measurement device of interest explicitly	3
	3	Specify the subject population of interest	3
	4	Specify the rater population of interest (if applicable)	4
	5	Describe what is already known about reliability and agreement and provide a rationale for the study (if applicable)	3
Methods	6	Explain how the sample size was chosen. State the determined number of raters, subjects/objects, and replicate observations	4
	7	Describe the sampling method	4
	8	Describe the measurement/rating process (e.g., time interval between repeated measurements, availability of clinical information, blinding)	4
	9	State whether measurements/ratings were conducted independently	4
	10	Describe the statistical analysis	4
Results	11	State the actual number of raters and subjects/objects that were included and the number of replicate observations that were conducted	5
	12	Describe the sample characteristics of raters and subjects (e.g., training, experience)	4-5
	13	Report estimates of reliability and agreement including measures of statistical uncertainty	6
Discussion	14	Discuss the practical relevance of results	8-11
Auxiliary material	15	Provide detailed results if possible (e.g., online)	6-11
